# Preparation of Desirable Porous Cell Structure Polylactide/Wood Flour Composite Foams Assisted by Chain Extender

**DOI:** 10.3390/ma10090999

**Published:** 2017-08-26

**Authors:** Youyong Wang, Yongming Song, Jun Du, Zhenhao Xi, Qingwen Wang

**Affiliations:** 1 Key Laboratory of Bio-Based Material Science and Technology (Ministry of Education), Northeast Forestry University, Harbin 150040, China; wangyouyong2018@yahoo.com (Y.W.); dujun9402@163.com (J.D.); 2 State Key Laboratory of Chemical Engineering, East China University of Science and Technology, Shanghai 200237, China; zhhxi@ecust.edu.cn; 3 College of Materials and Energy, South China Agricultural University, Guangzhou 510642, China; qwwang2006@sina.com

**Keywords:** chain extender, polylactide, wood flour, melt strength, crystallization, foams

## Abstract

Polylactide (PLA)/wood flour composite foam were prepared through a batch foaming process. The effect of the chain extender on the crystallization behavior and dynamic rheological properties of the PLA/wood flour composites were investigated as well as the crystal structure and cell morphology of the composite foams. The incorporation of the chain extender enhanced the complex viscosity and storage modulus of PLA/wood flour composites, indicating the improved melt elasticity. The chain extender also led to a decreased crystallization rate and final crystallinity of PLA/wood flour composites. With an increasing chain extender content, a finer and more uniform cell structure was formed, and the expansion ratio of PLA/wood flour composite foams was much higher than without the chain extender. Compared to the unfoamed composites, the crystallinity of the foamed PLA/wood flour composites was improved and the crystal was loosely packed. However, the new crystalline form was not evident.

## 1. Introduction

Diminishing petroleum resources and the increasingly serious “white pollution” due to petroleum-based plastics has spurred the development of environmentally-friendly materials. Bio-based and biodegradable polymers have been extensively studied [1,2,3,4]. These include polylactide (PLA), polyhydroxyalkanoates (PHAs), poly(butylene succinate) (PBS), and gamma-poly-l-glutamic acid (γ-PGA). PLA is one of the most promising polyesters for this purpose. On the one hand, PLA is a bio-based aliphatic polyester made from renewable resources. On the other, PLA exhibits excellent mechanical strength, biocompatibility, and biodegradability equivalent to, or even better than many petroleum-based polymers [5,6,7]. All of these properties make PLA, especially PLA foams [1,8,9,10,11,12,13,14], an alternative to petroleum-based polymer foams [15,16] in many applications ranging from daily necessities to the high added value products including food packaging, insulation panels, drug delivery, and biological scaffolds [6,7,17,18,19,20,21].

However, several drawbacks also limit the production and application of PLA products. These drawbacks include a low service temperature [22], low melt strength [20,23,24,25,26,27], and slow crystallization rate [28,29,30,31]. A relatively higher melt strength is normally required to sustain cell growth in the foaming processing, and the enhanced crystallization kinetics is needed during the final cell stabilization to avoid cell collapse and coalescence [28,30].

Natural fibers are abundant in nature, and are environmentally-friendly and renewable. These fibers can be combined with bio-based and biodegradable polymers to prepare new classes of fully bio-renewable composites. Natural fibers as well as inorganic nucleating agents [32,33,34,35,36] including talc, nanosilica, carbon nanotubes, and nanoclay have shown a significant effect on the crystallization kinetics of PLA. During the foaming processing, natural fibers provide more heterogeneous nucleation sites, which increases the cell density [36,37,38]. In addition to the crystallization, natural fibers can also improve the mechanical properties and thermostability of PLA [39,40].

However, it is still a challenge to prepare PLA/wood flour composite foams because of the low melt strength of PLA matrix. The melt strength of PLA can be improved by increasing the molecular weight and modifying the linear molecular structure of PLA through in situ ultraviolet-induced chain branching [41] and the incorporation of the chain extender [2,20,23,24,25,26,27,42,43,44,45,46]. In contrast, chain extender is more efficacious, and low chain extender content is needed for chain branching. Chain extender has been used in PLA foaming processing to introduce branched structures with significantly improved melt strength and melt viscoelasticity, which allow the polymer to withstand the triaxial deformation occurred during the cell growth process and optimize the cell structure [23,24,25,26,27,43]. However, the utilization of chain extender to assist the batch foaming of PLA/wood flour composites has not been well studied, especially in high wood flour loadings.

**Figure 1:**
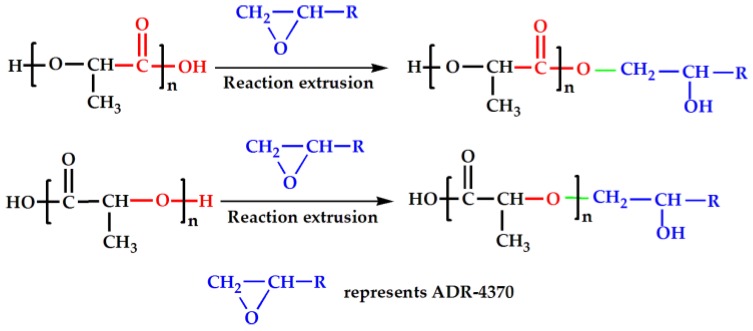
Schematic mechanism of chain extension with multi-functional extender.

In this paper, the styrene-acrylate oligomer multifunctional epoxide-based chain extender was used to modify the linear molecular structure and improve the molecular weight of PLA through reaction extrusion, thus obtaining the high melt strength PLA matrix, which in turn improved the foamability [27,47] of PLA/wood flour composites. The schematic mechanism of chain extension was described in Figure 1 [23,42]. The main objective was to prepare desirable porous cell structure PLA/wood flour composite foams with low apparent density, high cell density, better cell morphology, and a reasonably high expansion ratio by the introduction of a chain extender. The crystallization and rheological properties of PLA/wood flour composites were investigated as well.

## 2. Materials and Methods

### 2.1. Materials

PLA (3001D; MFI 24 g/10 min at 190 °C/2.16 kg, average molecular weight 72,000 g/mol) was obtained from Natureworks (Minnetonka, MN, USA). The wood flour (WF) with a particle size of about 150 μm was supplied from SipingCompany (Jilin, China). Talc (Mg_3_[Si_4_O_10_](OH)_2_; SiO_2_ content 61%; MgO content 31.5%) with a mean size 0.1 μm (Lingdong, China) was used as nucleating agent. CO_2_ with a purity of 99.95% was supplied as the blowing agent from Shanghai Gas Company (Shanghai, China). The chain extender (CE, ADR-4370; BASF, Ludwigshafen, Germany) is a multi-functional epoxide-based chain extender, composed of a styrene-acrylic oligomer with a number-averaged molecular weight (M_n_) around 6800 g/mol and a high number-average epoxy group functionality (f_n_) around 9 according to the supplier.

### 2.2. Preparation of PLA/Wood Flour Composite Sheets

Prior to preparing the PLA/wood flour composites, PLA and wood flour were oven-dried at 55 °C for 8 h and 103 °C for 12 h, respectively, to minimize their moisture levels. After being oven-dried, PLA (80 wt %), wood flour (20 wt %), CE (0, 0.5, 1.5, 2.5 wt %), talc (4 wt %), and lubricant (2 wt %) were mixed in a high-speed blender. The compounded mixtures were prepared using a parallel-rotating twin-screw extruder including seven heating zones at temperatures ranging from 155 to 180 °C, and the rotation speed was about 30 rpm. Finally, the extrudates were molded into composite sheets through hot pressing at 180 °C and 15 MPa constant pressure for 4 min at a thickness of 1.5~2 mm. The compression-molded sheets were cut into 10 × 20 mm^2^ rectangular specimens for the following batch foaming process.

### 2.3. Batch Foaming of PLA/Wood Flour Composites

**Figure 2:**
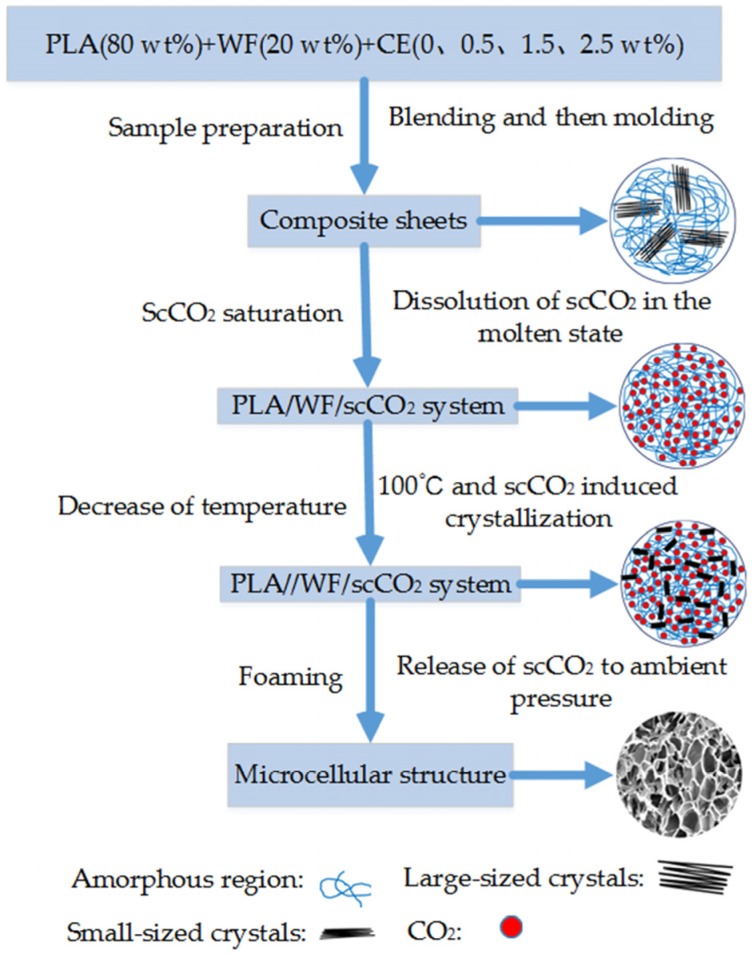
Process flow diagram for the preparation of Polylactide (PLA)/wood flour composite foams.

The batch foaming (Figure 2) experiments were carried out using a high-pressure vessel. The original weights of the aforementioned samples were measured by using a digital balance readable to 0.0001 g. The samples were placed into the high-pressure vessel and saturated with scCO_2_ at 16 MPa and 180 °C for about 20 min. The high-pressure vessel was then cooled to the foaming temperature of 100 °C, and the pressure dropped to 11 MPa correspondingly. The sample was saturated for another 20 min in this condition (100 °C and 11 MPa). In order to obtain the desirable porous cell structure, the high-pressure vessel was instantaneously depressurized to the atmospheric pressure to induce cell nucleation and growth. The foamed sample was taken out for cell solidification and shaping at atmospheric pressure and environment temperature.

### 2.4. Fourier Transform Infrared (FTIR) Spectrometry

FTIR spectra was implemented to detect the chemical grafting between PLA end-groups and the multifunctional epoxy group, using a Fourier transform infrared (FTIR) spectrometer (Nicolet Magna IR 560, Madison, WI, USA), with a scanning range of 4000~600 cm^−1^, a resolution of 4 cm^−1^, a scanning number of 32, and ATR mode was used for FTIR spectrometry. The specimens were prepared through hot pressing at a thickness of 1.5~2 mm and were dried before testing.

### 2.5. Rheological Properties

Dynamic shear rheological measurements were carried out using an AR2000ex rotational rheometer (TA Instruments, New Castle, PA, USA) in the oscillatory mode. A parallel-plate (25 mm diameter) geometry was selected for the frequency sweeps under 0.01% controlled strain. This strain value was first verified to be in the linear viscoelastic domain for all the samples. The angular frequencies were swept from 0.06283 to 628.3 rad·s^−1^ at 180 °C with a 2 mm gap.

### 2.6. Crystallization Analysis

Using a model Pyris Diamond device (PE Instruments, Waltham, MA, USA), differential scanning calorimetry (DSC) was done with a sample weight of 3~6 mg under the protection of nitrogen gas with a flow rate about 50 mL/min. The sample was rapidly heated from room temperature to 200 °C and then equilibrated at this temperature for 5 min to completely eliminate the previous thermal and stress histories. The sample was cooled to 25 °C at a rate of 10 °C /min. The sample was reheated to 200 °C at a rate of 10 °C /min. During all the heating and cooling processes, the DSC curves were recorded and analyzed using PE Universal software. The initial degree of crystallinity (*χ*_c_) of the sample was calculated as follows [24,25]:

(1) $$\chi_{c}=\frac{\Delta H_{m}}{\left(1-W_{t}\right)\Delta H_{0}}\times 100\%$$

where Δ*H_m_* is the melting enthalpy, *W_t_* is the mass fraction of substance except PLA, and Δ*H*_0_ is the melting enthalpy of 100% crystalline PLA (93.6 J/g) [22].

The kinetics of non-isothermal melt crystallization in the presence of CE were analyzed using the Avrami equation [28]:

(2) ln[−ln(1−X(t))]=nlnt+lnk

where *X*(*t*) is the relative crystallinity at crystallization time *t*, *k* is the crystallization kinetic constant for nucleation and growth rate, and *n* is the Avrami exponent reflecting the mechanisms of crystal nucleation and growth. By plotting ln[−ln(1 − *X*(*t*))] versus ln*t*, the Avrami exponent, *n*, and the logarithm of the kinetic constant, ln*k*, were determined.

The crystallization half-time (*t*_1/2_), which is the duration from the onset of the crystallization up to 50% completed crystallization, has been considered in the analysis of crystallization kinetics. The crystallization rate (*G*) is characterized as the reciprocal of *t*_1/2_. The crystallization half-time and rate can be calculated as shown in the following two equations:

(3) $$t_{1/2}={\left(\frac{\ln2}{k}\right)}^{1/n}$$

(4) $$G=\frac{1}{t_{1/2}}$$

### 2.7. Crystal Structure Analysis Using X-ray Diffraction (XRD)

XRD patterns were collected on a D/max2200 X-ray diffractometer (Rigaku, Tokyo, Japan) with the sample exposed to a high resolution Cu Kα radiation source at room temperature, 40 KV, and 30 mA. The scanned range for all samples was 10~30° (2θ), with a step size of 2°/min and time per step of 2 s.

### 2.8. Foam Characterization

The densities of the unfoamed and foamed samples were measured according to ASTM D792-13 (Standard test methods for density of plastics by water displacement) at room temperature (25 °C), which involved measuring the weights of at least five randomly selected samples for each formulation in the air and the increased weights when put into the water. Density was determined using the following equation:

(5) $$\rho =\frac{m}{\Delta m}\rho_{0}$$

where *ρ* and *ρ*_0_ is the density of the sample and water, respectively; *ρ*_0_ is considered as the density of water at room temperature (1 g/cm^−3^), m is the weight in air, and Δ*m* is the increased weight when the sample is put into water.

The expansion ratio (*ER*) of the sample was then evaluated using the following equation [36]:

(6) $$ER=\frac{\rho_{polymer}}{\rho_{foam}}$$

where *ρ_polymer_* and *ρ_foam_* is the calculated apparent densities of unfoamed and foamed samples, respectively, determined by the method above.

The porous cell morphologies of the foamed samples were observed by scanning electron microscopy (SEM) using a Quanta 200 microscope (FEI, Hillsboro, OR, USA). The samples were immersed in liquid nitrogen for 5 min and then fractured. The fractured surface was coated with a thin layer of gold. SEM images containing more than 200 cells were analyzed using ImageJ processing software (NIH, Bethesda, MD, USA). The cell density of the foamed sample was then measured using the following equation:

(7) $$N_{0}={\left(\frac{nM^{2}}{A}\right)}^{\frac{3}{2}}$$

where *n* is the number of bubbles in the micrograph, *A* is the area of the micrograph, and *M* is the magnification factor of the micrograph, and *N*_0_ is the cell density of foamed sample. In the cell density calculation, the sample was assumed to have been free of unfoamed skin.

## 3. Results and Discussion

### 3.1. FTIR Analysis

**Figure 3:**
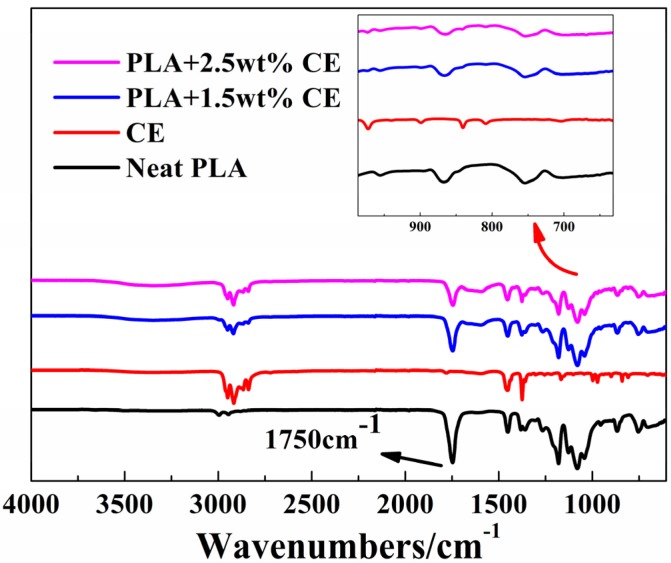
FTIR spectra for Neat PLA, CE and PLA modified by CE.

To clarify the interaction between the epoxy group and carboxyl group, the functional properties of neat PLA, CE, and PLA modified by CE were supported by ATR-FTIR spectra, as shown in Figure 3. It can be noted that the weak characteristic bands at 900 cm^−1^ for CE were associated with the asymmetric stretching of the epoxy group [48]. In addition, for the PLA/CE system, the disappearance of the characteristic bands for epoxy group meant that the epoxy group vanished, indicating that the chain extension reaction was carried out in the reaction extrusion. Furthermore, PLA and modified PLA showed an adsorption band at 1750 cm^−1^ ascribed to the stretching vibration of the carbonyl group, and the intensity of this band decreased with the incorporation of CE, which was due to the interaction of the epoxy group and carboxyl group. On the basis of the chain branching by CE, the molecular weight of PLA will be improved [23,49,50], and the linear molecular structure of PLA will be turned into a branched molecular structure, thus contributing to the high melt strength and improved foamability.

### 3.2. Rheological Properties

**Figure 4:**
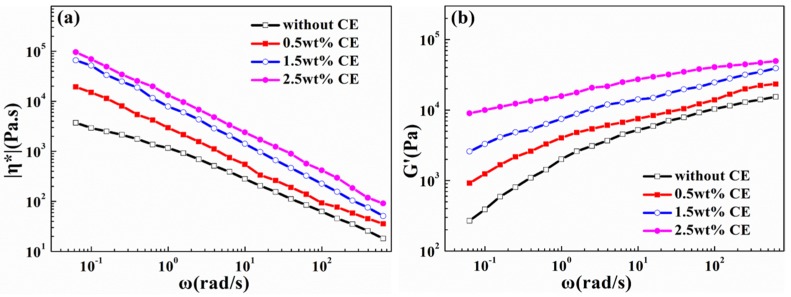
(**a**) Complex viscosity (η*) as a function of angular frequency (ω) for PLA/WF/Chain Extender (CE) at 180 °C, 0.01% and 0.06283~628.3 rad.s^−1^; (**b**) Storage modulus (G’) as a function of angular frequency (ω) PLA/WF/CE at 180 °C, 0.01% and 0.06283~628.3 rad.s^−1^.

Complex viscosity is crucial in the foaming process, which can improve the cell rupture and cell collapse phenomenon. The complex viscosity of the ideal foam system should be controlled in a certain range. As shown in Figure 4a, all of the samples in the measured frequency range displayed typical shear thinning behaviors. In the low frequency, with increasing CE content, the viscosity of PLA/wood flour composites increased, due to the formation of the branched chain structure and the improved molecular weight after chain extension reaction, which enhances chain entanglement and increases the polymer flow resistance. However, after CE content reached 1.5 wt %, the complex viscosity increased by a relative low degree, which implies a threshold of CE concentration [23,26].

Figure 4b depicts the storage modulus of the composites as a function of angular frequency. The storage modulus reflects the elasticity of composites, which has a close relationship with the melt strength and is an indication of the resistance for the melt extension. As shown in Figure 4b, the storage modulus increased with an increasing CE content, resulting from the existence of branched structure that could store deformation energy [23]. Moreover, in the low-frequency region, the composites become less frequency-dependent, indicating that the viscoelastic response is changing from a pseudoplastic-like to pseudosolid-like behavior. That is, over a range of frequencies the balance between viscous and elastic properties is retained because of the formation of gel-like pseudostructures [43,44].

### 3.3. Crystallization Behavior

#### 3.3.1. Effect of Foaming on Crystallinity

**Figure 5:**
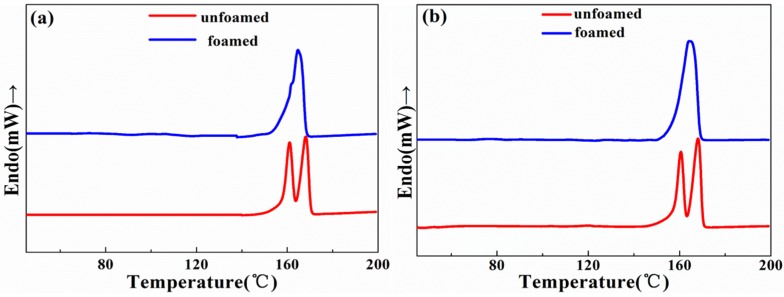
Differential scanning calorimetry (DSC) thermograms for foamed and unfoamed samples without CE (**a**) and in the presence of 2.5 wt % CE (**b**).

The effect of foaming on the crystallization behavior of PLA/wood flour composites was investigated. As shown in Figure 5, the unfoamed sample displayed low and high melting temperature peaks regardless of whether it contained CE or not. The double melting peak phenomenon is common for the semi-crystallization polymer, which results from the melt-reorganization of the less ordered crystals [28]. However, the foamed samples show only a single broader melting peak, which indicates the generation of more different crystalline structures through the foaming induced chain overlap. The foamed samples exhibited an obviously higher crystallinity as compared to the unfoamed samples (Table 1). The higher crystallinity derived from the plasticization effect of scCO_2_, which can improve the mobility of PLA molecular chains and allow the orderly arrangement of a large number of chains in the lattice. In addition, coupling with the dissolved scCO_2_, the biaxial stretching that occurs in the stage of cell expansion and growth can induce a high degree of crystallization due to the strain-induced crystallization [28], both of which improve the final crystallinity.

#### 3.3.2. Non-Isothermal DSC Thermograms and Avrami Analysis

**Figure 6:**
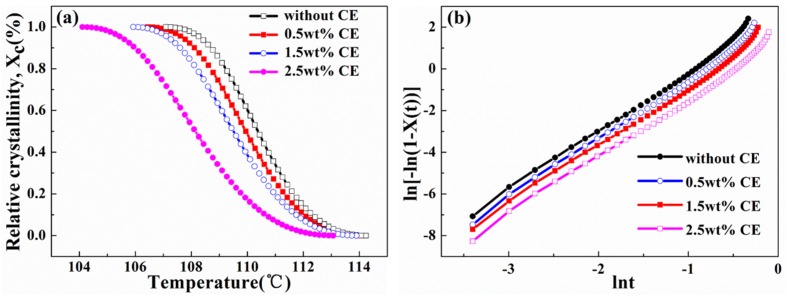
Relative crystallinity versus temperature plot (**a**) and corresponding Avrami double-log plot (**b**) for unfoamed composites with CE.

Figure 6a,b display a plot of relative crystallinity versus temperature and the corresponding Avrami double-log plot for composites with CE, respectively. As illustrated in Figure 6a, the incorporation of CE decreased the crystallinity of the PLA/wood flour composites, due to the introduction of branched structure, which greatly hindered the movement of PLA molecular chains and prevent the dense packing of chains [27,28], so that the crystallinity decreased. As calculated in Table 2, Avrami exponent *n* was close to 3, indicating that the crystals grew mainly as a two-dimensional crystal plate and three-dimensional spherulite in non-isothermal crystallization [28,29]. Crystallization rate constant *k* decreased with increasing CE content. The molecular structure is the root of the different crystallization rate, because the molecular chain activation energy demanded to spread into the crystal phase structure varies with the molecular structure. The increase of the CE content improves the branching degree of PLA [26]. Chain segment diffusion and the chain arrangement were restrained during the non-isothermal crystallization, so increased CE loadings lower the crystallization kinetics.

### 3.4. Crystal Structure Analysis Using XRD

**Figure 7:**
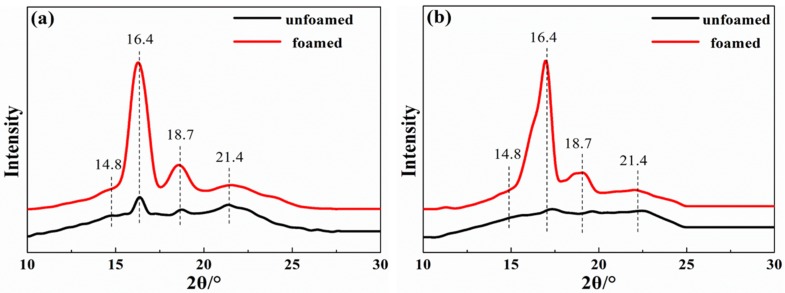
X-ray diffraction patterns of the unfoamed and foamed samples without CE (**a**) and in the presence of 2.5 wt % CE (**b**).

The XRD profiles of unfoamed and foamed PLA/wood flour composites without CE and in the presence of 2.5 wt % CE are illustrated in Figure 7. Both of the unfoamed and foamed samples exhibited diffraction peaks at around 2θ = 14.8°, 16.4°, 18.7°, and 21.4°. These diffraction peaks are in agreement with the α-crystal form of PLA, and are attributed to the reflections of (010), (110/200), (203), (015), and (016) planes [36], respectively. However, as compared to the unfoamed composites in the Figure 7a,b, the intensity of the diffraction peaks of foamed composites increased and the peak width increased, especially at 2θ = 16.4° and 18.7°. The peak width can be correlated with the packing of the crystalline structure, where a wider peak reflects a loosely packed crystalline structure, and a narrow peak represents a closely packed structure. The peak intensity represents the relative crystallinity. The shear stress and biaxial stretching rearranged and aligned the PLA molecular chains into an ordered structure (crystals), thereby enhancing the degree of crystallinity, consistent with the results in Table 1. At the same time, the dissolved scCO_2_ and foaming process increased the free volume of the PLA chains, which resulted in the formation of a large number of small-size crystals. These results indicate that the foaming process affected the crystallinity of PLA, but did not change the crystalline form.

### 3.5. Apparent Density, Expansion Ratio and Cell Density

**Figure 8:**
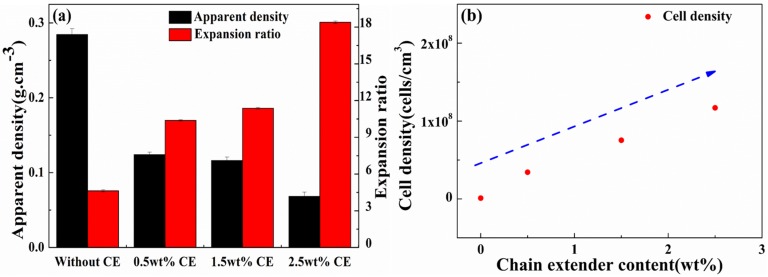
Foam characterization of PLA/Wood flour composite foams: Apparent density, Expansion ratio, (**a**) and Cell density (**b**) as a function of CE content.

Figure 8a depicts the apparent density and expansion ratio of the foamed samples as a function of the CE content. The expansion ratio increased with an increasing CE content. In contrast, the apparent density decreased. When the CE content was 2.5 wt %, the expansion ratio increased by up to 18 times, with a low apparent density of 0.0682 g/cm^3^. In the absence of CE, linear PLA has a poor melt strength and typically does not exhibit any strain-hardening behavior [23,26]. A further drawback of PLA is that it has poor thermal stability and can undergo chain-scission during processing [26]. During the foaming process, the melt cannot tolerate the strain induced by the cell expansion and growth, resulting in the cell coalescence, then cell rupture, and then finally cell collapse. The addition of CE introduces the branched structure, which improves the melt strength of composites. Cell coalescence as well as cell collapse phenomenon is reduced, and most of the bubbles during cell growth can be preserved, which in turn increases the expansion ratio and reduces the apparent density of composite foams.

Corresponding cell density of the PLA/wood flour composite foams in the presence of CE is shown in Figure 8b. Cell density mainly depends on the number of nucleation sites and the solubility of scCO_2_. The content of wood flour and Talc is the same. However, the cell density almost increases in a linear fashion with the CE content, showing the quantitative effect of CE on the efficiency of global nucleation. Cross-linked sites that couple with the PLA end group can promote the heterogeneous nucleation, so increased CE content increases the nucleation sites [23,26,41], as a result, the cell nucleation density becomes larger. Moreover, the CE decreases the crystallinity of PLA/wood flour composites (shown in Table 1), improving the solubility of scCO_2_ in the PLA resin [51], thus increasing the cell density.

### 3.6. Cell Micrographs and Cell Size Distribution

**Figure 9:**
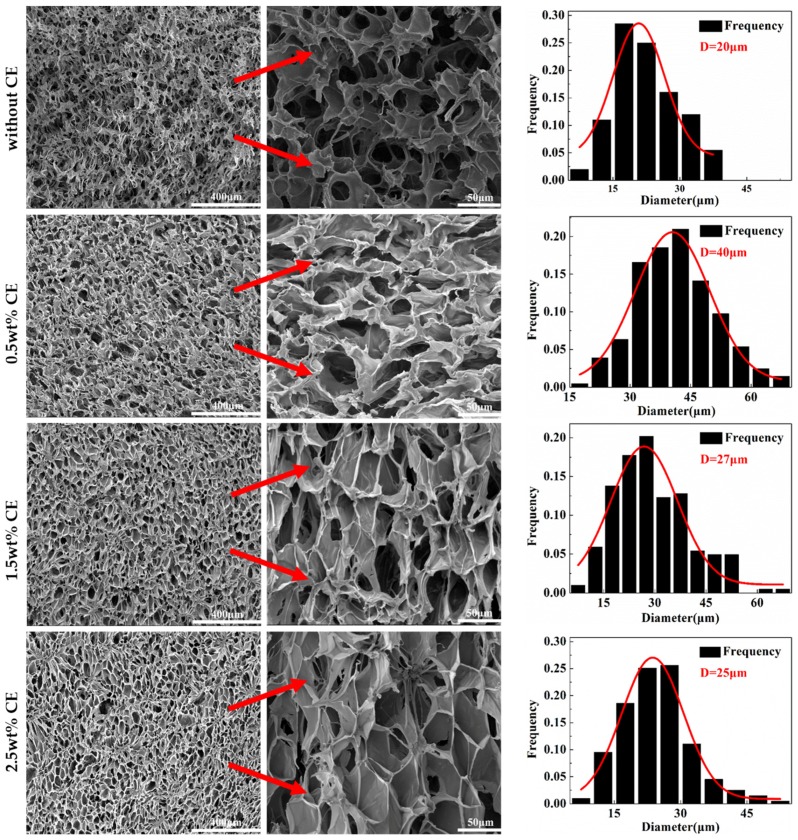
SEM micrographs of fracture surface of the polylactide/wood flour composite foams and the corresponding cell size distribution in the presence of various CE content.

Figure 9 displays SEM micrographs of the cross-section fractured by liquid nitrogen, and the corresponding cell size distribution of the PLA/wood flour composite foams. It was observed that CE showed a significant effect on the cell structure of the composite foams. For the sample without CE, the cell rupture phenomenon was serious, which resulted in high open-cell ratio due to the poor melt strength. As the CE content increased to 0.5 wt %, the cell wall became thinner, cell morphology was obviously improved due to the improved melt elasticity. However, cells still displayed an abnormal shape because the chain extension reaction could not be carried out adequately at the low CE loadings, which could not create strain-hardening. Further increasing the extender content from 1.5 wt % to 2.5 wt %, the cell morphology was much better. The closed cells take much account, and the cell shape is a regular polygon. Chain extension reaction was well carried out when enough CE was added into the system. Microscopically, most of the PLA molecular chains have a branched structure (shown in Figure 1), leading to the higher melt strength and strain-hardening behavior during the foaming process. The biaxial extension stress around each cell is uniform, so the foamability is markedly improved and the final cell adopts a regular shape.

All of the cell size distributions of the foams obeyed Gaussian distribution. From the fitted curve, it was evident that smaller cells constituted a greater proportion of samples lacking CE, due to the low viscosity and low melt strength, which resulted in the formation of small-sized cells with an interconnected structure. Larger cells began to appear after CE was introduced to the system because of the increased expansion ability. Moreover, the cell diameter decreased by a large degree and the cell size distribution became significantly narrowed with increasing CE content, which was due to the improved melt strength through the adequate chain extension reaction. When the CE content was increased up to 2.5 wt %, the average cell diameter was 25 μm, and the cell size distribution was narrow.

## 4. Conclusions

In this study, the effects of CE on the crystallization behavior, dynamic rheological property, and foamability of the PLA/wood flour composites were investigated. CE improved the complex viscosity and storage modulus in the low frequency, the storage modulus reflects the elasticity of composites, which has a close relationship with the melt strength, thus the improved melt strength and elasticity, which in turn promoted the foamability of PLA/wood flour composites. Moreover, the crystallinity as well as the crystallization rate of the composites declined because of the formation of a branched structure. In addition, the crystallinity of PLA/wood flour composites increased after the foaming processing, but the foaming behavior did not induce a new crystalline form, which attributes to the plasticization effect of scCO_2_ and the strain-induced crystallization in the foaming process. Compared to the PLA/wood flour composite foams lacking CE, 2.5 wt % CE significantly improved the porous cell morphology. The expansion ratio increased up to 18 times, the apparent density was 0.0682 g/cm^3^, the average cell size was 25 μm, the cell density reached 1.2 × 10^8^ cells/cm^3^ with a more uniform cell size distribution, and a more closed-cell structure was obtained. As a result, desirable porous cell structure PLA/wood flour composite foams were prepared, which would contribute to the subsequent continuous production of PLA/wood flour composite foams.

## Figures and Tables

**Table 1:** Crystallinity and melting parameters of foamed and unfoamed samples without CE and in the presence of 2.5 wt % CE.

Samples		*T*_L_/(°C)	*T*_H_/(°C)	*X*_C_/(%)
Without CE	unfoamed	160.9	168.1	40.65
	foamed	—	164.8	41.07
2.5 wt % CE	unfoamed	160.6	168.1	36.20
	foamed	—	164.1	46.74

***T*_L_**: Low melting peak temperature of PLA; ***T*_H_**: High melting peak temperature of PLA; ***X*_C_**: crystallinity of PLA.

**Table 2:** Crystallization half-time, crystallization rate, and Avrami model’s parameters of unfoamed composites with CE.

Samples	*t*_1/2_ (min)	*G* (min^−1^)	*n*	ln*k*	*k*
Without CE	0.38	2.63	2.93	2.44	11.45
0.5 wt % CE	0.41	2.43	2.90	2.22	9.16
1.5 wt % CE	0.43	2.33	2.87	2.03	7.61
2.5 wt % CE	0.48	2.08	2.84	1.70	5.45

*t*_1/2_: crystallization half-time; *G*: crystallization rate; *n*: Avrami exponent; ln*k*: the logarithm of the kinetic constant; *k*: crystallization kinetic constant.
